# (2-Amino­phen­yl)[(5*S*)-5-hydr­oxy-3,5-dimethyl-4,5-dihydro-1*H*-pyrazol-1-yl]methanone

**DOI:** 10.1107/S1600536809026294

**Published:** 2009-07-11

**Authors:** Mohammad Arfan, M. Nawaz Tahir, Rasool Khan, Sumbal Saba, Mohammad S. Iqbal

**Affiliations:** a Institute of Chemical Sciences, University of Peshawar, Peshawar 25120, Pakistan; b Department of Physics, University of Sargodha, Sargodha, Pakistan; c Department of Chemistry, Government College University, Lahore, Pakistan

## Abstract

In the mol­ecule of the title compound, C_12_H_15_N_3_O_2_, the pyrazole ring is oriented at a dihedral angle of 49.64 (6)° with respect to the benzene ring. Intra­molecular O—H⋯O, N—H⋯O and C—H⋯O inter­actions result in the formation of a trifurcated hydrogen bond. In the crystal structure, inter­molecular N—H⋯O and O—H⋯N hydrogen bonds link the mol­ecules, forming a network structure.

## Related literature

For general background to the diverse medical potential of pyrazoles and their modified forms, see: Gürsoy *et al.* (2000 ▶); Lynch & McClenaghan (2005 ▶). For the biological activity of pyrazolopyrimidines, see: Shaabani *et al.* (2009 ▶). For synthetic procedures for the preparation of the 4,5-dihydro­pyrrazole nucleus bearing various functionalities on the ring, see: Bahreni *et al.* (2009 ▶); Kumarasinghe *et al.* (2009 ▶); Liu *et al.* (2009 ▶); Lynch & McClenaghan (2005 ▶). For bond-length data, see: Allen *et al.* (1987 ▶).
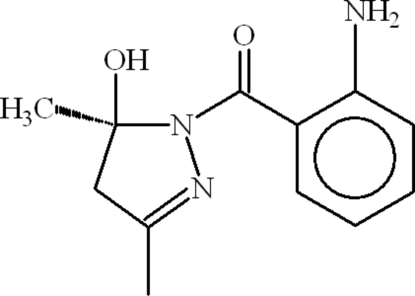


## Experimental

### 

#### Crystal data

C_12_H_15_N_3_O_2_
*M* *_r_* = 233.27
Orthorhombic, 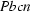

*a* = 23.5705 (7) Å
*b* = 11.3547 (4) Å
*c* = 9.1848 (3) Å
*V* = 2458.18 (14) Å^3^
*Z* = 8
Mo *K*α radiation
μ = 0.09 mm^−1^
*T* = 296 K
0.28 × 0.20 × 0.18 mm

#### Data collection

Bruker Kappa APEXII CCD diffractometer
Absorption correction: multi-scan (*SADABS*; Bruker, 2005 ▶) *T*_min_ = 0.975, *T*_max_ = 0.984
14464 measured reflections
3163 independent reflections
2438 reflections with *I* > 2σ(*I*)
*R*_int_ = 0.022

#### Refinement

*R*[*F* ^2^ > 2σ(*F* ^2^)] = 0.044
*wR*(*F* ^2^) = 0.126
*S* = 1.04
3163 reflections
165 parameters
H atoms treated by a mixture of independent and constrained refinement
Δρ_max_ = 0.30 e Å^−3^
Δρ_min_ = −0.20 e Å^−3^

### 

Data collection: *APEX2* (Bruker, 2007 ▶); cell refinement: *SAINT* (Bruker, 2007 ▶); data reduction: *SAINT*; program(s) used to solve structure: *SHELXS97* (Sheldrick, 2008 ▶); program(s) used to refine structure: *SHELXL97* (Sheldrick, 2008 ▶); molecular graphics: *ORTEP-3 for Windows* (Farrugia, 1997 ▶) and *PLATON* (Spek, 2009 ▶); software used to prepare material for publication: *WinGX* (Farrugia, 1999 ▶) and *PLATON*.

## Supplementary Material

Crystal structure: contains datablocks global, I. DOI: 10.1107/S1600536809026294/hk2735sup1.cif

Structure factors: contains datablocks I. DOI: 10.1107/S1600536809026294/hk2735Isup2.hkl

Additional supplementary materials:  crystallographic information; 3D view; checkCIF report

## Figures and Tables

**Table 1:** Hydrogen-bond geometry (Å, °)

*D*—H⋯*A*	*D*—H	H⋯*A*	*D*⋯*A*	*D*—H⋯*A*
N1—H1*A*⋯O1	0.868 (18)	2.181 (18)	2.8351 (16)	131.9 (15)
N1—H1*A*⋯O1^i^	0.868 (18)	2.380 (17)	2.9882 (15)	127.4 (15)
N1—H1*B*⋯O1^ii^	0.892 (18)	2.166 (18)	3.0277 (16)	162.4 (16)
O2—H2⋯O1	0.82	2.52	2.9527 (14)	114
O2—H2⋯N1^i^	0.82	2.17	2.9741 (16)	165
C6—H6⋯N3	0.93	2.60	2.9499 (18)	103
C12—H12*C*⋯O1	0.96	2.56	3.0548 (19)	112

Symmetry codes: (i) 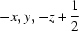; (ii) 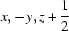.

**Fig. 1.**
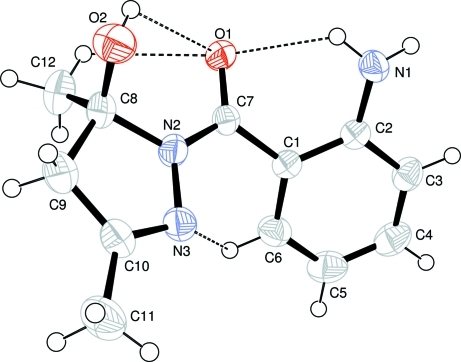
The molecular structure of the title molecule, with the atom-numbering scheme. Displacement ellipsoids are drawn at the 50% probability level. Hydrogen bonds are shown as dashed lines.

**Fig. 2.**
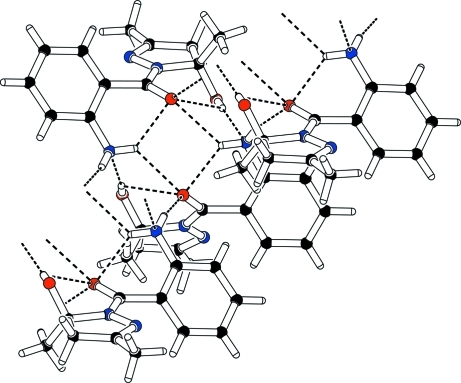
A partial packing diagram of the title compound. Hydrogen bonds are shown as dashed lines.

## supplementary crystallographic information

### Comment

The chemistry of pyrazoles and their modified forms are of great interest to synthetic medicinal researchers because of their diverse medicinal potential reported in the literature including analgesic (Gürsoy *et al.* 2000), antiinflammatory (Lynch & McClenaghan, 2005) and other therapeutic functions. Pyrazolopyrimidines have shown wide ranging biological activities including vasodilatory, antihypertensive, antiepileptic, anxiolytic, antidepressant and oncolytic while pyrazolopyridines have exhibited anxiolytic, xanthine oxidase inhibitors, cholesterol formation inhibitors and potential remedies for Alzheimer's disease, gastrointestinal diseases, anorexia nervosa and infertlity (Shaabani *et al.*, 2009). Although synthetic procedures for the preparation of 4,5-dihydropyrrazole nucleus bearing various functionalities on the ring have appeared so far in the literature (Liu *et al.*, 2009; Kumarasinghe *et al.*, 2009; Bahreni *et al.*, 2009; Lynch & McClenaghan, 2005), we report for first time the synthesis of 3,4-dimethyl-4,5-dihydro-1*H*-pyrazol-5-ol nucleus through a silica gel catalyzed one-pot two components cyclocondensation.

In the molecule of the title compound (Fig. 1), the bond lengths (Allen *et al.*,  1987) and angles are within normal ranges. Rings A (C1-C6) and B (N1/N3/C8-C10) are, of course, planar, and they are oriented at a dihedral angle of A/B = 49.64 (6)°. Intramolecular O-H···O, N-H···O, C-H···N and C-H···O interactions (Table 1) result in the formations of non-planar six-membered rings having twisted conformations: C (N2/N3/C1/C6/C7/H6), D (O1/N1/C1/C2/C7/H1A), E (O1/O2/N2/C7/C8/H2) and F (O1/N2/C7/C8/C12/H12C).

In the crystal structure, intermolecular N-H···O and O-H···N hydrogen bonds (Table 1) link the molecules to form a polymeric network (Fig. 2), in which they may be effective in the stabilization of the structure.

### Experimental

For the preparation of the title compound, 2-aminobenzohydrazide (0.15 g, 1 mmol) and a slight excess of acetylacetone (2 ml) over silica gel catalyst were stirred at room temperature for 12 h. After completion of reaction, the product was extracted with acetone. The solvent was evaporated under reduced pressure and recrystallized from ethanol (yield; 57%, m. p. 402-403 K).

### Refinement

H atoms (for NH_2_) were located in a difference Fourier map and their coordinates were refined. The remaining H atoms were positioned geometrically with O-H = 0.82 Å (for OH) and C-H = 0.93, 0.97 and 0.96 Å for aromatic, methylene and methyl H atoms, respectively and constrained to ride on their parent atoms, with U_iso_(H) = xU_eq_(C,N,O), where x = 1.5 for methyl H and x = 1.2 for all other H atoms.

### Crystal data

**Table:**

C_12_H_15_N_3_O_2_	*F*(000) = 992
*M**_r_* = 233.27	*D*_x_ = 1.261 Mg m^−^^3^
Orthorhombic, *P**b**c**n*	Mo *K*α radiation, λ = 0.71073 Å
Hall symbol: -P 2n 2ab	Cell parameters from 3163 reflections
*a* = 23.5705 (7) Å	θ = 3.0–28.7°
*b* = 11.3547 (4) Å	µ = 0.09 mm^−^^1^
*c* = 9.1848 (3) Å	*T* = 296 K
*V* = 2458.18 (14) Å^3^	Prism, yellow
*Z* = 8	0.28 × 0.20 × 0.18 mm

### Data collection

**Table:**

Bruker Kappa APEXII CCD diffractometer	3163 independent reflections
Radiation source: fine-focus sealed tube	2438 reflections with *I* > 2σ(*I*)
graphite	*R*_int_ = 0.022
Detector resolution: 7.40 pixels mm^-1^	θ_max_ = 28.7°, θ_min_ = 3.0°
ω scans	*h* = −31→30
Absorption correction: multi-scan (*SADABS*; Bruker, 2005)	*k* = −15→15
*T*_min_ = 0.975, *T*_max_ = 0.984	*l* = −11→12
14464 measured reflections	

### Refinement

**Table:**

Refinement on *F*^2^	Primary atom site location: structure-invariant direct methods
Least-squares matrix: full	Secondary atom site location: difference Fourier map
*R*[*F*^2^ > 2σ(*F*^2^)] = 0.044	Hydrogen site location: inferred from neighbouring sites
*wR*(*F*^2^) = 0.126	H atoms treated by a mixture of independent and constrained refinement
*S* = 1.04	*w* = 1/[σ^2^(*F*_o_^2^) + (0.0599*P*)^2^ + 0.624*P*] where *P* = (*F*_o_^2^ + 2*F*_c_^2^)/3
3163 reflections	(Δ/σ)_max_ < 0.001
165 parameters	Δρ_max_ = 0.30 e Å^−^^3^
0 restraints	Δρ_min_ = −0.20 e Å^−^^3^

### Special details

**Table:**

Geometry. Bond distances, angles *etc*. have been calculated using the rounded fractional coordinates. All su's are estimated from the variances of the (full) variance-covariance matrix. The cell e.s.d.'s are taken into account in the estimation of distances, angles and torsion angles
Refinement. Refinement of *F*^2^ against ALL reflections. The weighted *R*-factor *wR* and goodness of fit *S* are based on *F*^2^, conventional *R*-factors *R* are based on *F*, with *F* set to zero for negative *F*^2^. The threshold expression of *F*^2^ > σ(*F*^2^) is used only for calculating *R*-factors(gt) *etc*. and is not relevant to the choice of reflections for refinement. *R*-factors based on *F*^2^ are statistically about twice as large as those based on *F*, and *R*- factors based on ALL data will be even larger.

### Fractional atomic coordinates and isotropic or equivalent isotropic displacement parameters (Å^2^)

**Table:**

	*x*	*y*	*z*	*U*_iso_*/*U*_eq_	
O1	0.05818 (4)	0.11538 (8)	0.14455 (10)	0.0359 (3)	
O2	0.00495 (5)	0.31866 (9)	−0.00220 (13)	0.0505 (3)	
N1	0.06263 (5)	0.10115 (11)	0.45251 (14)	0.0379 (4)	
N2	0.09915 (5)	0.28723 (10)	0.08904 (12)	0.0351 (3)	
N3	0.13457 (5)	0.38251 (10)	0.12462 (13)	0.0378 (3)	
C1	0.13799 (5)	0.16794 (10)	0.28915 (14)	0.0299 (3)	
C2	0.11982 (5)	0.11841 (10)	0.42083 (14)	0.0301 (3)	
C3	0.16031 (6)	0.09259 (12)	0.52774 (16)	0.0398 (4)	
C4	0.21706 (6)	0.11357 (14)	0.50342 (19)	0.0500 (5)	
C5	0.23509 (6)	0.16073 (16)	0.3730 (2)	0.0536 (5)	
C6	0.19585 (5)	0.18704 (13)	0.26614 (17)	0.0418 (4)	
C7	0.09593 (5)	0.18893 (11)	0.17072 (13)	0.0294 (3)	
C8	0.06207 (6)	0.31127 (13)	−0.04089 (15)	0.0395 (4)	
C9	0.08216 (7)	0.43582 (15)	−0.08055 (19)	0.0521 (5)	
C10	0.12563 (6)	0.46354 (12)	0.03036 (16)	0.0401 (4)	
C11	0.15629 (9)	0.57783 (15)	0.0355 (2)	0.0616 (6)	
C12	0.07320 (8)	0.22171 (17)	−0.15852 (18)	0.0565 (6)	
H1A	0.0417 (7)	0.0897 (15)	0.376 (2)	0.0454*	
H1B	0.0558 (7)	0.0465 (16)	0.520 (2)	0.0454*	
H2	−0.00781	0.25221	0.00998	0.0606*	
H3	0.14873	0.06089	0.61624	0.047 (4)*	
H4	0.24343	0.09581	0.57548	0.058 (5)*	
H5	0.27347	0.17474	0.35709	0.065 (5)*	
H6	0.20811	0.21786	0.17776	0.046 (4)*	
H9A	0.05099	0.49149	−0.07606	0.0624*	
H9B	0.09834	0.43743	−0.17763	0.0624*	
H11A	0.18200	0.57798	0.11659	0.0924*	
H11B	0.17722	0.58855	−0.05315	0.0924*	
H11C	0.12945	0.64081	0.04652	0.0924*	
H12A	0.05694	0.24852	−0.24844	0.0848*	
H12B	0.11338	0.21170	−0.17043	0.0848*	
H12C	0.05638	0.14785	−0.13165	0.0848*	

### Atomic displacement parameters (Å^2^)

**Table:**

	*U*^11^	*U*^22^	*U*^33^	*U*^12^	*U*^13^	*U*^23^
O1	0.0378 (5)	0.0361 (5)	0.0338 (5)	−0.0054 (4)	−0.0037 (4)	−0.0016 (4)
O2	0.0426 (5)	0.0468 (6)	0.0621 (7)	0.0037 (4)	−0.0006 (5)	0.0086 (5)
N1	0.0352 (6)	0.0442 (7)	0.0342 (6)	−0.0015 (5)	−0.0001 (4)	0.0097 (5)
N2	0.0418 (6)	0.0332 (5)	0.0303 (6)	−0.0038 (4)	−0.0047 (4)	0.0028 (5)
N3	0.0469 (6)	0.0309 (5)	0.0357 (6)	−0.0048 (4)	0.0010 (5)	−0.0009 (5)
C1	0.0318 (6)	0.0255 (5)	0.0325 (7)	0.0012 (4)	−0.0017 (5)	−0.0014 (5)
C2	0.0334 (6)	0.0232 (5)	0.0337 (7)	0.0022 (4)	−0.0022 (5)	−0.0013 (5)
C3	0.0456 (7)	0.0372 (7)	0.0365 (7)	0.0037 (5)	−0.0085 (6)	0.0047 (6)
C4	0.0417 (8)	0.0535 (9)	0.0549 (10)	0.0050 (6)	−0.0177 (7)	0.0016 (7)
C5	0.0308 (7)	0.0637 (10)	0.0662 (11)	−0.0003 (7)	−0.0051 (7)	0.0003 (9)
C6	0.0345 (6)	0.0464 (8)	0.0444 (8)	−0.0019 (5)	0.0035 (6)	0.0020 (6)
C7	0.0325 (5)	0.0290 (6)	0.0266 (6)	0.0018 (4)	0.0025 (4)	−0.0023 (5)
C8	0.0427 (7)	0.0426 (7)	0.0331 (7)	0.0011 (5)	−0.0046 (5)	0.0088 (6)
C9	0.0589 (9)	0.0494 (9)	0.0479 (9)	−0.0038 (7)	−0.0038 (7)	0.0177 (7)
C10	0.0480 (7)	0.0343 (7)	0.0380 (7)	0.0001 (5)	0.0079 (6)	0.0024 (6)
C11	0.0822 (12)	0.0384 (8)	0.0642 (11)	−0.0139 (8)	0.0031 (10)	0.0090 (8)
C12	0.0705 (10)	0.0657 (11)	0.0334 (8)	0.0035 (9)	−0.0054 (7)	−0.0011 (8)

### Geometric parameters (Å, °)

**Table:**

O1—C7	1.2438 (15)	C8—C9	1.535 (2)
O2—C8	1.3950 (18)	C8—C12	1.507 (2)
O2—H2	0.8200	C9—C10	1.479 (2)
N1—C2	1.3929 (17)	C10—C11	1.486 (2)
N2—C7	1.3470 (17)	C3—H3	0.9300
N2—C8	1.5042 (18)	C4—H4	0.9300
N2—N3	1.4051 (16)	C5—H5	0.9300
N3—C10	1.2808 (18)	C6—H6	0.9300
N1—H1B	0.892 (18)	C9—H9A	0.9700
N1—H1A	0.868 (18)	C9—H9B	0.9700
C1—C2	1.4009 (18)	C11—H11A	0.9600
C1—C6	1.3970 (17)	C11—H11B	0.9600
C1—C7	1.4909 (17)	C11—H11C	0.9600
C2—C3	1.4004 (19)	C12—H12A	0.9600
C3—C4	1.377 (2)	C12—H12B	0.9600
C4—C5	1.379 (2)	C12—H12C	0.9600
C5—C6	1.381 (2)		
			
O1···O2	2.9527 (14)	C11···H9B^vii^	2.9700
O1···N1	2.8351 (16)	H1A···O1	2.181 (18)
O1···C12	3.0548 (19)	H1A···C7	2.541 (18)
O1···N1^i^	2.9882 (15)	H1A···O1^i^	2.380 (17)
O1···N1^ii^	3.0277 (16)	H1A···H2^i^	2.2700
O2···N1^i^	2.9741 (16)	H1B···H3	2.3700
O2···O1	2.9527 (14)	H1B···O1^v^	2.166 (18)
O1···H1A^i^	2.380 (17)	H2···O1	2.5200
O1···H12C	2.5600	H2···C7	2.9500
O1···H1B^ii^	2.166 (18)	H2···H12C	2.3200
O1···H1A	2.181 (18)	H2···N1^i^	2.1700
O1···H2	2.5200	H2···H1A^i^	2.2700
O2···H12A^iii^	2.8300	H3···H1B	2.3700
O2···H9A^iv^	2.6300	H3···C1^v^	3.0600
N1···O1	2.8351 (16)	H4···N3^viii^	2.9200
N1···O1^i^	2.9882 (15)	H4···C11^viii^	3.1000
N1···O2^i^	2.9741 (16)	H4···H6^viii^	2.5800
N1···O1^v^	3.0277 (16)	H6···N2	2.8100
N3···C6	2.9499 (18)	H6···N3	2.6000
N1···H2^i^	2.1700	H6···C4^vi^	3.0600
N1···H12C^v^	2.9300	H6···H4^vi^	2.5800
N2···H6	2.8100	H9A···O2^iv^	2.6300
N3···H6	2.6000	H9B···H12A	2.4400
N3···H4^vi^	2.9200	H9B···H12B	2.5900
N3···H9B^vii^	2.8700	H9B···N3^ix^	2.8700
C6···N3	2.9499 (18)	H9B···C10^ix^	2.9800
C12···O1	3.0548 (19)	H9B···C11^ix^	2.9700
C1···H3^ii^	3.0600	H11B···C6^ix^	3.0700
C2···H11C^vii^	2.9800	H11C···C2^ix^	2.9800
C4···H6^viii^	3.0600	H12A···H9B	2.4400
C6···H11B^vii^	3.0700	H12A···O2^iii^	2.8300
C7···H2	2.9500	H12B···H9B	2.5900
C7···H12C	2.9700	H12C···O1	2.5600
C7···H1A	2.541 (18)	H12C···C7	2.9700
C10···H9B^vii^	2.9800	H12C···H2	2.3200
C11···H4^vi^	3.1000	H12C···N1^ii^	2.9300
			
C8—O2—H2	109.00	N3—C10—C11	121.72 (14)
N3—N2—C7	122.82 (11)	C9—C10—C11	123.00 (14)
N3—N2—C8	112.96 (10)	N3—C10—C9	115.26 (13)
C7—N2—C8	124.02 (11)	C2—C3—H3	120.00
N2—N3—C10	107.35 (11)	C4—C3—H3	120.00
C2—N1—H1B	114.7 (11)	C3—C4—H4	120.00
C2—N1—H1A	113.7 (12)	C5—C4—H4	120.00
H1A—N1—H1B	110.8 (16)	C4—C5—H5	120.00
C2—C1—C6	119.43 (12)	C6—C5—H5	120.00
C2—C1—C7	119.39 (10)	C1—C6—H6	120.00
C6—C1—C7	120.93 (12)	C5—C6—H6	120.00
N1—C2—C1	122.19 (11)	C8—C9—H9A	111.00
C1—C2—C3	118.76 (11)	C8—C9—H9B	111.00
N1—C2—C3	118.92 (12)	C10—C9—H9A	111.00
C2—C3—C4	120.80 (14)	C10—C9—H9B	111.00
C3—C4—C5	120.49 (14)	H9A—C9—H9B	109.00
C4—C5—C6	119.66 (13)	C10—C11—H11A	109.00
C1—C6—C5	120.84 (14)	C10—C11—H11B	109.00
N2—C7—C1	120.09 (11)	C10—C11—H11C	109.00
O1—C7—N2	119.28 (11)	H11A—C11—H11B	109.00
O1—C7—C1	120.62 (11)	H11A—C11—H11C	109.00
O2—C8—C9	107.62 (12)	H11B—C11—H11C	109.00
O2—C8—C12	113.04 (13)	C8—C12—H12A	109.00
O2—C8—N2	111.69 (11)	C8—C12—H12B	109.00
N2—C8—C9	100.15 (11)	C8—C12—H12C	109.00
N2—C8—C12	110.20 (12)	H12A—C12—H12B	109.00
C9—C8—C12	113.45 (13)	H12A—C12—H12C	109.00
C8—C9—C10	104.26 (13)	H12B—C12—H12C	109.00
			
C7—N2—N3—C10	−176.72 (12)	C7—C1—C2—C3	176.17 (11)
C8—N2—N3—C10	−1.71 (15)	C2—C1—C6—C5	−1.8 (2)
N3—N2—C7—O1	170.03 (11)	C7—C1—C6—C5	−176.01 (14)
N3—N2—C7—C1	−11.16 (18)	C2—C1—C7—O1	−40.35 (17)
C8—N2—C7—O1	−4.42 (19)	C2—C1—C7—N2	140.86 (12)
C8—N2—C7—C1	174.39 (11)	C6—C1—C7—O1	133.87 (13)
N3—N2—C8—O2	−112.23 (12)	C6—C1—C7—N2	−44.92 (17)
N3—N2—C8—C9	1.48 (14)	N1—C2—C3—C4	−176.93 (13)
N3—N2—C8—C12	121.25 (13)	C1—C2—C3—C4	−1.1 (2)
C7—N2—C8—O2	62.70 (16)	C2—C3—C4—C5	0.1 (2)
C7—N2—C8—C9	176.42 (12)	C3—C4—C5—C6	0.0 (2)
C7—N2—C8—C12	−63.81 (17)	C4—C5—C6—C1	0.9 (2)
N2—N3—C10—C9	1.18 (17)	O2—C8—C9—C10	116.07 (13)
N2—N3—C10—C11	179.96 (13)	N2—C8—C9—C10	−0.73 (14)
C6—C1—C2—N1	177.60 (12)	C12—C8—C9—C10	−118.11 (14)
C6—C1—C2—C3	1.86 (18)	C8—C9—C10—N3	−0.24 (18)
C7—C1—C2—N1	−8.09 (18)	C8—C9—C10—C11	−179.00 (14)

Symmetry codes: (i) −*x*, *y*, −*z*+1/2; (ii) *x*, −*y*, *z*−1/2; (iii) −*x*, *y*, −*z*−1/2; (iv) −*x*, −*y*+1, −*z*; (v) *x*, −*y*, *z*+1/2; (vi) −*x*+1/2, −*y*+1/2, *z*−1/2; (vii) *x*, −*y*+1, *z*+1/2; (viii) −*x*+1/2, −*y*+1/2, *z*+1/2; (ix) *x*, −*y*+1, *z*−1/2.

### Hydrogen-bond geometry (Å, °)

**Table:**

*D*—H···*A*	*D*—H	H···*A*	*D*···*A*	*D*—H···*A*
N1—H1A···O1	0.868 (18)	2.181 (18)	2.8351 (16)	131.9 (15)
N1—H1A···O1^i^	0.868 (18)	2.380 (17)	2.9882 (15)	127.4 (15)
N1—H1B···O1^v^	0.892 (18)	2.166 (18)	3.0277 (16)	162.4 (16)
O2—H2···O1	0.82	2.52	2.9527 (14)	114
O2—H2···N1^i^	0.82	2.17	2.9741 (16)	165
C6—H6···N3	0.93	2.60	2.9499 (18)	103
C12—H12C···O1	0.96	2.56	3.0548 (19)	112

Symmetry codes: (i) −*x*, *y*, −*z*+1/2; (v) *x*, −*y*, *z*+1/2.
